# Comparative Analysis of Gene Expression Profiles of Human Dental Fluorosis and Kashin-Beck Disease

**DOI:** 10.1038/s41598-017-18519-z

**Published:** 2018-01-09

**Authors:** Qiang Zhang, Jing Ma, Haiqing Liu, Duolong He, Lilin Chen, Haikun Wu, Hong Jiang, Qing Lu, Shenglu Bai

**Affiliations:** Qinghai Institute For Endemic Disease Prevention and Control, Qinghai, 811602 China

## Abstract

To explore the pathologies of Kashin-Beck disease (KBD) and KBD accompanied with dental fluorosis (DF), we conducted a comparative analysis of gene expression profiles. 12 subjects were recruited, including 4 KBD patients, 4 patients with KBD and DF and 4 healthy subjects. Genome-wide expression profiles from their peripheral blood mononuclear cells were evaluated by customized oligonucleotide microarray. R programming software was used for the microarray data analysis followed by functional enrichment analysis through KOBAS. Several potential biomarkers were identified, and quantitative real-time reverse transcription–polymerase chain reaction (qRT-PCR) was used for their validation. In this study, 28 genes and 8 genes were found to be up- and down-regulated respectively in KBD patients compared with health subjects. In patients with KBD and DF, we obtained 10 up-regulated and 3 down-regulated genes compared with health controls. Strikingly, no differential expression gene (DEG) was identified between the two groups of patients. A total of 10 overlaps (DUSP2, KLRF1, SRP19, KLRC3, CD69, SIK1, ITGA4, ID3, HSPA1A, GPR18) were obtained between DEGs of patients with KBD and patients with KBD and DF. They play important roles in metabolism, differentiation, apoptosis and bone-development. The relative abundance of 8 DEGs, i.e. FCRL6, KLRC3, CXCR4, CD93, CLK1, GPR18, SRP19 and KLRF1, were further confirmed by qRT-PCR analysis.

## Introduction

Kashin-Beck disease (KBD) is an endemic and chronic osteoarthropathy which mostly occurs in children between the ages of 3 and 12 in Asia^1^. The primary pathogenesis of KBD include chondrocyte degeneration, necrosis, progressive loss of articular cartilage and so on, which should result in arthrosis deformities in adult^2,3^. In China, over 30 million people are at risk of KBD. There have been three etiological hypotheses of KBD, biology-earth-chemistry hypothesis (selenium deficiency), water organic compound poisoning hypothesis, and food fungi toxin poisoning hypothesis, but the accurate etiology of KBD is still unknown^4–6^.

Li *et al*. showed that low fluorine in the living environment caused an internal metabolic disorder, leading to pathological change of KBD^7^. In addition, fluorosis and KBD are endemic diseases, which are closely associated with geology, geochemistry, hydrology and etc^8^. Dental fluorosis (DF) is an early manifestation of fluorosis, which is caused by the excessive input of fluoride. There are three types of endemic fluorosis, including drinking-water type, coal-burning type and drinking-tea type. It was reported that water type fluorosis is the most common type in China, especially DF in children^9,10^.

It has been reported that genetic factors play important roles in the pathogenesis of KBD and DF. Extensive genetic studies have been conducted to identify susceptibility genes for KBD and DF, and several valuable biomarkers have been obtained, such as HLA-DRB1, ITPR2, ADAM12, ameloblastin gene, and ER Rsa I. However, the mechanism of KBD and DF remains unclear. The variations of KBD and DF explained by the identified loci were limited, suggesting the existence of undiscovered genetic variants associated with KBD and DF.

The purpose of this study is to explore the common pathogenesis and the underlying molecular functions of KBD and DF. In this study, Affymetrix PrimeView™ Human Gene Expression Array was used for the quantification of genome-wide expression profiles from peripheral blood mononuclear cells of patients with KBD, KBD and DF, as well as healthy controls, for the comparative analysis. Functional enrichment analysis identified important processes related to the progression of KBD and DF. Several biomarkers were further confirmed by qRT-PCR. This should be helpful for the understanding of mechanisms of KBD and DF and the development of novel drugs and therapeutic methods.

## Material and Methods

### Study Population

Study samples consists of 12 Han Chinese subjects, including 4 patients with KBD, 4 patients with DF and KBD and 4 healthy controls. Patients with KBD were diagnosed as grade II or grade III according to the clinical criteria (diagnostic code GB16395-1996). Donors of dental fluorosis were diagnosed with the modified Dean classification, which is national diagnosis standard and experimental test for fluoride (WS/T 208–2011). The subjects with osteoarthritis (OA), Rheumatoid arthritis (RA) and other skeletal diseases were excluded. All subjects were matched based on age and gender. This study was approved by Qinghai Institute for Endemic Disease Prevention and Control and Medical ethics committee of Qinghai Institute for Endemic Disease Prevention and Control. All participants signed inform-consent documents. This study was carried by *Qinghai provincial health and Family Planning Commission of science and Technology Education Department memo (2008) No. 6*.

### RNA Extraction

The peripheral blood were collected and stored at −80 °C. Blood samples needed to thaw at room temperature for 2 hours before RNA extraction. Total RNA was extracted with Agilent Total RNA Isolation Mini kit (Agilent Technologies, Santa Clara, CA) following the manufacturer’s instructions. The RNA amount was normalized using The Human-Actin Competitive PCR Set (Takara Bio, Kyoto, Japan). To check the integrity of the total RNA, 60 ng (normalized value) denatured total RNA was subjected to 1% agarose gel electrophoresis, and dyed with ethidium bromide. Extracted RNA was stored at −80 °C until cDNA synthesis.

### Microarray Hybridization

Total RNA was reverse-transcribed into complementary DNA (cDNA), and then transcribed into cRNA and labled with Cy-Dye using Amino Allyl MessageAmp aRNA Kit (Ambion) following the manufacturer’s instructions. Thereafter, 0.5 μg of each labeled cRNA was purified separately and then mixed with hybridization buffer before being applied on the microarray. The hybridization solution was prepared with the *In Situ* Hybridization Plus kit (Agilent Technologies), and hybridization was performed in the hybridization chamber (Gene-Machines, San Carlos, CA, USA). Conditions of hybridization and washing were in accordance with the manufacturer’s recommendations (Agilent Technologies).

### Analysis of Microarray Data

Affymetrix mRNA microarray were analyzed and transferred into CEL signal files using Affymetrix® GeneChip® Command Console® Software. A possible dye bias in the results was eliminated using an algorithm for the same extraction software (Extraction 9.3 Software, Agilent) that involves normalization factors (global normalization, location normalization). Normalized expression values was used for the identification of DEGs with limma package^11^ of R based on the thresholds of fold change >2 or <0.5 and FDR adjusted p-value < 0.05. For the exploration of processes involved in the development of KBD and DF, we also conducted functional enrichment analysis for DEGs through KOBAS^12^ with the thresholds of p-value < 0.05.

### Quantitative Real-Time Reverse Transcription PCR

Total RNA was prepared for qRT-PCR. These RNA samples were transformed into complementary DNA (cDNA) using Superscript II reverse transcriptase (Invitrogen, Carlsbad, CA) and random primers. qRT-PCR was operated using the ABI 7500 Real-Time PCR system (Applied Biosys-tems, Foster City, CA) according to the manufacturer’s specification.

To validate the microarray results, 8 significant differentially expressed genes were selected for parallel qRT-PCR analysis, including FCRL6, KLRC3, CXCR4, CD93, CLK1, GPR18, SRP1, KLRF1. Data were analyzed with the 2−ΔΔCt method using GAPDH as internal control.

### Statistical analysis

R version3.2.2 was used for all of the statistical analysis. The relative mRNA level in the qPCR analysis was represented by mean ± SD of the three replicates, and p-value < 0.05 was considered as statistical significant.

## Results

### Microarray Data Analysis

**Figure 1:**
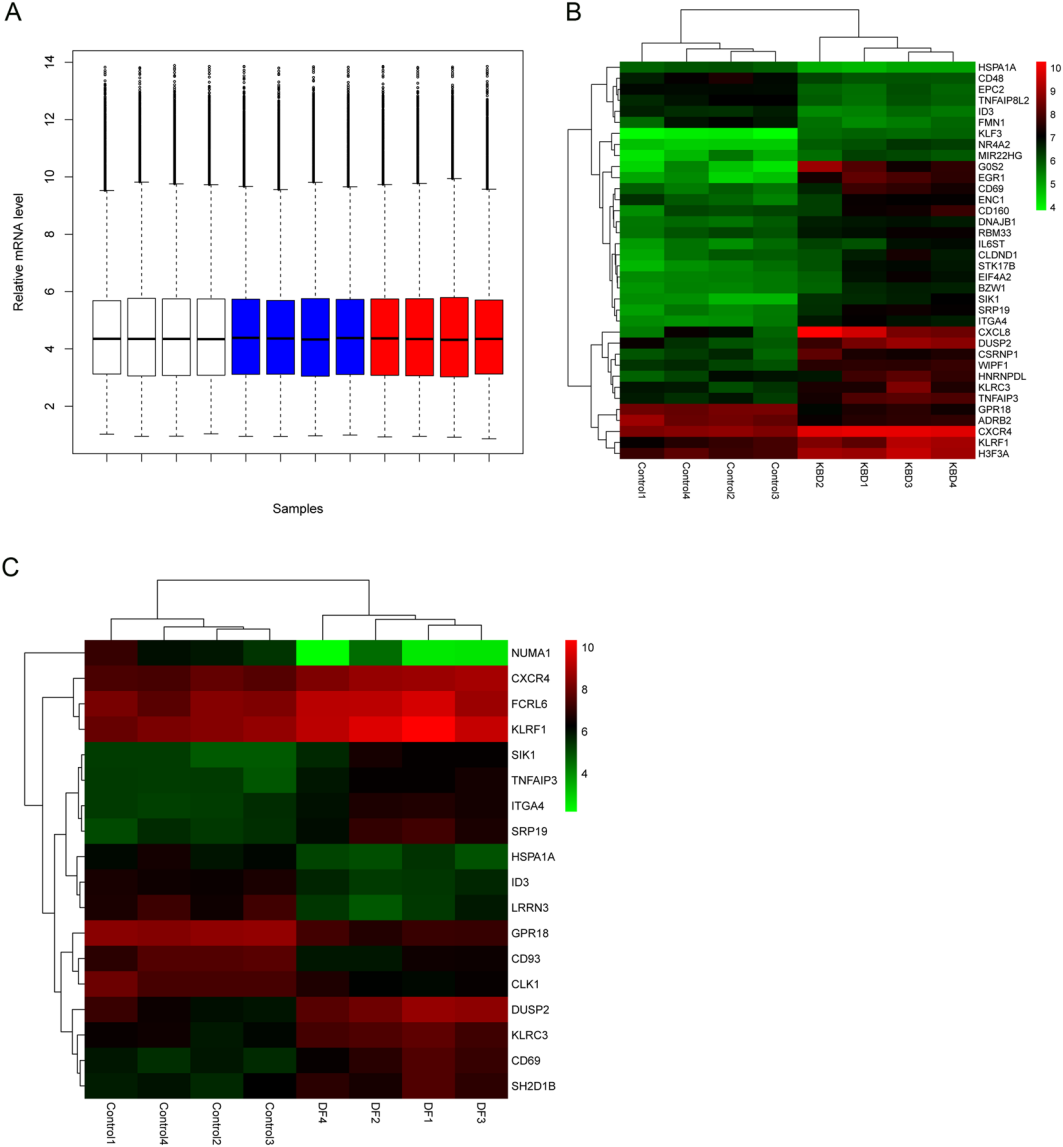
Microarray analysis. (**A**) Relative mRNA level of all samples after normalization. White boxes are healthy controls, blue boxes are KBD samples and red boxes are KBD with DF samples. (**B**) Two-way hierarchical clustering of expression profiles and samples of healthy controls and KBD samples. (**C**) Two-way hierarchical clustering of expression profiles and samples of healthy controls and KBD with DF samples.

The GeneChip® PrimeView™ Human Gene Expression Array provides comprehensive coverage of the human genome in a cartridge array. Figure 1A illustrates the overall expression profiles in all samples. Comparable expression levels were obtained after normalization, which should be suitable for the following analysis. With the specified thresholds, 28 genes at higher level and 8 genes at lower level in KBD patients compared with controls were obtained. Tables 1 and 2 shows the up- and down-regulated genes and their enriched functions respectively. For patients with KBD and DF, we identified 11 up-regulated and 7 down-regulated genes compared with healthy controls. Tables 3 and 4 is the up- and down-regulated genes and their enriched functions respectively. Figure 1B,C illustrates the heatmap of DEGs in KBD patients and KBD and DF patients respectively in which green and red represents low and high expression level. A total of 10 overlaps, including 7 up-regulated (DUSP2, KLRF1, SRP19, KLRC3, CD69, SIK1 and ITGA4) and 3 down-regulated (ID3, HSPA1A and GPR18) genes, were identified between the two lists of DEGs. Strikingly, no gene was found to be significantly differential expression between patients with KBD and patients with DF.

**Table 1:** List of genes differentially expressed in KBD *(up-regulated genes).

Category	Gene Name	Public ID	Gene Symbol	Fold Change
apoptosis	G0/G1 switch 2	NM_015714	G0S2	10.1038
	nuclear receptor subfamily 4, group A, member 2	NM_006186	NR4A2	3.9593
	salt-inducible kinase 1	NM_173354	SIK1	3.1203
	early growth response 1	NM_001964	EGR1	8.6298
	interleukin 6 signal transducer	NM_001190981	IL6ST	2.1023
metabolism	chemokine (C-X-C motif) ligand 8	NM_000584	CXCL8	7.4122
	Kruppel-like factor 3 (basic)	NM_016531	KLF3	3.3914
	signal recognition particle 19 kDa	NM_001204193	SRP19	3.0827
	cysteine-serine-rich nuclear protein 1	NM_033027	CSRNP1	2.7026
signal transduction	dual specificity phosphatase 2	NM_004418	DUSP2	4.3656
	CD69 molecule	NM_001781	CD69	3.1153
	killer cell lectin-like receptor subfamily C, member 3	NM_002261	KLRC3	2.5084
	eukaryotic translation initiation factor 4A2	NM_001967	EIF4A2	2.3559
development corrlelated	integrin, alpha 4	NM_000885	ITGA4	2.8429
	chemokine (C-X-C motif) receptor 4	NM_001008540	CXCR4	2.6099
cystoskeleton and cell movement	serine/threonine kinase 17b	NM_004226	STK17B	2.5863
	WAS/WASL interacting protein family, member 1	NM_001077269	WIPF1	2.5435
	ectodermal-neural cortex 1 (with BTB domain)	NM_001256574	ENC1	2.0278
bone resorption	tumor necrosis factor, alpha-induced protein 3	NM_001270507	TNFAIP3	2.5231
membrane protein	killer cell lectin-like receptor subfamily F, member 1	NM_001291822	KLRF1	2.4904
	CD160 molecule	NM_007053	CD160	2.2795
	claudin domain containing 1	NM_001040181	CLDND1	2.1154
DNA modification	H3 histone, family 3A	NM_002107	H3F3A	2.3112
RNA process	heterogeneous nuclear ribonucleoprotein D-like	NM_001207000	HNRNPDL	2.2607
	basic leucine zipper and W2 domains 1	NM_001207067	BZW1	2.039
miscellaneous	MIR22 host gene (non-protein coding)	NM_032895	MIR22HG	2.2257
	DnaJ (Hsp40) homolog, subfamily B, member 1	NM_006145	DNAJB1	2.0591
	RNA binding motif protein 33	NM_001008408	RBM33	2.0549

**Table 2:** List of genes differentially expressed in KBD *(down-regulated genes).

Category	Gene Name	Public ID	Gene Symbol	Fold Change
metabolism	enhancer of polycomb homolog 2	NM_015630	EPC2	0.4687
	inhibitor of DNA binding 3	NM_002167	ID3	0.4964
signal transduction	G protein-coupled receptor 18	NM_001098200	GPR18	0.4933
cystoskeleton and cell	heat shock 70 kDa protein 1 A	NM_005345	HSPA1A	0.4949
movement	formin 1	NM_001103184	FMN1	0.4882
membrane protein	CD48 molecule	NM_001256030	CD48	0.4984
	adrenoceptor beta 2	NM_000024	ADRB2	0.4836
miscellaneous	tumor necrosis factor, alpha-induced protein 8-like 2	NM_024575	TNFAIP8L2	0.4928

**Table 3:** List of genes differentially expressed in KBD with DF (up-regulated genes).

Category	Gene Name	Public ID	Gene Symbol	Fold Change
development correlated	dual specificity phosphatase 2	NM_004418	DUSP2	3.4894
	integrin, alpha 4	NM_000885	ITGA4	2.261
menbrane protein	killer cell lectin-like receptor subfamily F, member 1	NM_001291822	KLRF1	2.6744
	signal recognition particle 19 kDa	NM_001204193	SRP19	2.6216
	Fc receptor-like 6	NM_001004310	FCRL6	2.098
signal transduction	killer cell lectin-like receptor subfamily C, member 3	NM_002261	KLRC3	2.639
	CD69 molecule	NM_001781	CD69	2.4183
	salt-inducible kinase 1	NM_173354	SIK1	2.3002
apoptosis	tumor necrosis factor, alpha-induced protein 3	NM_001270507	TNFAIP3	2.1203
cystoskeleton and cell movement	chemokine (C-X-C motif) receptor 4	NM_001008540	CXCR4	2.0905
miscellaneous	SH2 domain containing 1B	NM_053282	SH2D1B	2.182

**Table 4:** List of genes differentially expressed in KBD with DF (down-regulated genes).

Category	Gene Name	Public ID	Gene Symbol	Fold Change
development correlated	inhibitor of DNA binding 3	NM_002167	ID3	0.4673
	G protein-coupled receptor 18	NM_001098200	GPR18	0.3675
menbrane protein	CD93 molecule	NM_012072	CD93	0.4206
cystoskeleton and cell movement	nuclear mitotic apparatus protein 1	NM_001286561	NUMA1	0.1127
metabolism	heat shock 70 kDa protein 1 A	NM_005345	HSPA1A	0.4804
	leucine rich repeat neuronal 3	NM_001099658	LRRN3	0.3146
miscellaneous	CDC-like kinase 1	NM_001024646	CLK1	0.4413

### qRT-PCR Validation of Microarray Data

**Figure 2:**
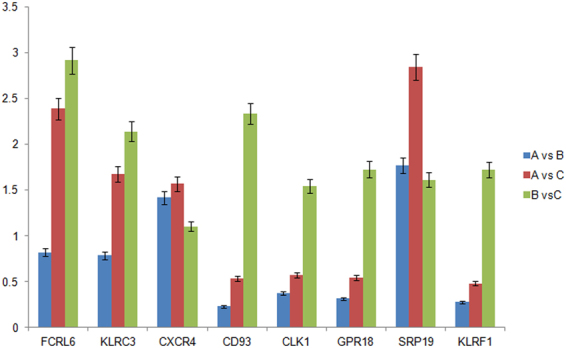
Histogram showing the ratio of expression levels of the 8 selected genes in three groups, as analyzed by quantitative real-time reverse transcription–polymerase chain reaction (PCR) (n = 12). Bars show the standard deviations. (**A**), (**B**) and (**C**) is KBD samples, KBD with DF samples and healthy subjects respectively.

8 genes were further verified by qRT-PCR. The results of qRT-PCR experiment were consistent with microarray analysis. According to qRT-PCR results, the expression levels of KLRC3, KLRF1, SRP19 and CXCR4 were higher in KBD and KBD with DF than controls, while expression levels of CLK1 and GPR18 were lower in both KBD and KBD with DF samples compared with healthy controls (shown in Fig. 2). Besides, FCRL6 was at higher level and CD93 was at lower level only in patients with KBD and DF.

## Discussion

KBD and DF are complex diseases that determined by genetic to a large extent. In this study, we conducted a comparative analysis of gene expression profiles for patients with KBD, KBD and DF, and healthy controls using Affymetrix PrimeView™ Human Gene Expression Array. qRT-PCR was used to validate the oligonucleotide array data. Based on the gene ontology enrichment analysis, we divided these genes into different categories, including metabolism, apoptosis, cytoskeleton, signal transduction and bone development-related genes. Cartilage damage is the main characteristics of pathological changes in KBD, including necrosis in deeper articular cartilage, excessive apoptosis of chondrocytes, extracellular matrix degradation and so on^13–15^. Endemic fluorosis is a chronic systemic diseases, characterized with lumbocrural pain, anchylosis, bone-deformity. It is necessary to study bone development-related genes of KBD and DF. Significant differences in gene expression pattern were observed between patients with KBD and healthy controls, as well as between patients with KBD and DF and healthy controls. Ten genes (7 up-regulated and 3 down-regulated) were found to be consistently differentially expressed in KBD and KBD with DF samples, which contains several bone development-related genes, such as DUSP2, ITGA4, ID3, GPR18, and they should provide valuable information for further understanding of KBD and DF.

In mammalian cells, the dual-specificity phosphatase (DUSP) family is responsible for the dephosphorylation of threonine and tyrosine residues. Hamamura *et al*. showed that Dusp2 could suppress inflammation in antibody-induced arthritis in a mouse model through down-regulating inflammatory signs^16^. Besides, DUSP2 is involved in response to oxidative stress and apoptotic signaling, which play important roles in the development of KBD. Yin *et al*. reported that DUSP2 transcription was induced in response to oxidative stress, causing p53-dependent apoptosis^17^. Moreover, DUSP2 involved in the process of salvianolic acid a (SAA) effects rat cardiomyocytes by mediating regulation of the ERK1/2/JNK pathway^18^.

It was reported that the expression of integrin was associated with the osteoarthritis severity, especially ITGA4 (integrinα4). Becerril M *et al*. declared that ITGA4 played an important role in the loss of proteoglycans and clusters formation at OA early stages^19^. Proteoglycans, the main component of extracellular matrix of cartilage, were associated with articular cartilage metabolism in patients with KBD^1,20^. Consistent with the previous studies, we also identified ITGA4 as an important biomarker in the pathology of KBD.

Inhibitor of DNA Binding 3 (ID3), a transcription factor involved in the development of T cell and growth inhibition of a B cell progenitors, plays an important role in controlling cell cycle progression^21–23^. Thornemo *et al*. reported that ID3 is important for chondrocyte differentiation and ID proteins are expressed in a lot of cell types and decrease in various cell lines during differentiation^24^. Here, ID3 was also proved to be down-regulated in KBD, as well as KBD and DF samples, which should indicate its roles in the progression of KBD and DF.GPR18, one of the orphan G protein-coupled receptors, has been found to be a receptor for endogenous lipid neurotransmitters. Ramos *et al*. reported that GPR18 was differentially expressed in osteoarthritis patients^25^. Takenouchi *et al*. declared that GPR18 involved in the regulation of apoptosis^26^, and apoptosis plays an important role on pathological process of KBD, so GPR18 might contribute the development of KBD.

In summary, we conducted a comparative analysis of gene expression profiles to explore the common pathogenesis and the underlying molecular functions between KBD and DF. Significant differences in gene expression pattern were found between KBD, KBD with DF samples and healthy controls. Our results should provide novel insights for further study of the molecular mechanism of KBD and DF. While, further studies should be conducted to confirm our findings.
